# tRF‐15:31‐Arg‐CCT‐3‐1 Improves Diabetic Kidney Disease by Reducing AATF and Alleviating Mesangial Cell Oxidative Stress and Inflammation

**DOI:** 10.1002/iid3.70561

**Published:** 2026-10-19

**Authors:** Beibei Ning, Chan Huang, Xuewei Liu, Yunyang Qiao, Jialing Ji, Huimin Shi, Gaoting Qu, Yajie Zhao, Weihua Gan, Aiqing Zhang

**Affiliations:** ^1^ Department of Pediatrics The Second Affiliated Hospital of Nanjing Medical University Nanjing China; ^2^ Department of Nephrology and Immunology Children's Hospital of Soochow University Suzhou China; ^3^ Department of Pediatrics The Fourth Affiliated Hospital of Nanjing Medical University Nanjing China

**Keywords:** AATF, diabetic kidney disease, inflammation, oxidative stress, tRF‐15:31‐Arg‐CCT‐3‐1

## Abstract

**Background:**

Diabetic kidney disease (DKD), a primary contributor to end‐stage renal disease, involves a complex interplay between oxidative stress and inflammation. Given the reported involvement of apoptosis‐antagonizing transcription factor (AATF) in cellular stress and inflammatory responses, we investigated whether the anti‐inflammatory effects of tRF‐15:31‐Arg‐CCT‐3‐1 (tRF‐15:31) are mediated through the regulation of AATF expression.

**Methods:**

The expression and biological effects of tRF‐15:31 were investigated in streptozotocin (STZ)‐induced DKD mice and high‐glucose (HG)‐treated renal mesangial cells. The involvement of AATF was examined through tRF‐15:31 and AATF overexpression and siRNA‐mediated knockdown of AATF.

**Results:**

Reduced expression of tRF‐15:31 was observed in both STZ‐induced DKD mice and HG‐treated renal mesangial cells and was associated with aggravated intrarenal inflammation and oxidative stress. Overexpression of tRF‐15:31 in diabetic mice significantly reduced serum creatinine levels and 24‐h urinary protein excretion and alleviated renal pathological injury. Both in vivo and in vitro, tRF‐15:31 overexpression markedly downregulated pro‐inflammatory and oxidative stress markers, including IL‐6, MCP‐1, TNF‐α, and malondialdehyde (MDA), while upregulating antioxidant factors such as glutathione (GSH) and glutathione peroxidase 4 (GPX4). AATF expression was significantly elevated in STZ‐induced DKD mice, whereas this upregulation was markedly suppressed by tRF‐15:31 overexpression. In HG‐treated mesangial cells, co‐overexpression of tRF‐15:31 and AATF reversed the protective effects of tRF‐15:31 overexpression alone. Furthermore, siRNA‐mediated knockdown of AATF significantly attenuated HG‐induced inflammation and oxidative stress.

**Conclusion:**

Collectively, these findings suggest that tRF‐15:31 attenuates inflammation and oxidative stress in the STZ‐induced DKD model, at least in part through regulation of AATF expression. These findings provide additional mechanistic evidence for tRF‐mediated regulation in DKD and support further investigation of the biological significance of the tRF‐15:31–AATF regulatory relationship.

## 1 Introduction

Diabetic kidney disease (DKD) is a major diabetes‐related microvascular disorder and currently accounts for a substantial proportion of end‐stage renal disease (ESRD) worldwide [1, 2]. Approximately 40% of individuals with diabetes progress to ESRD [3], imposing a substantial economic burden worldwide. DKD pathogenesis is intricate and variable; accordingly, new DKD treatments are urgently required. Increasing evidence indicates that chronic inflammation and immune dysregulation play central roles in the initiation and progression of DKD, linking metabolic stress to sustained renal injury [4, 5].

The development of DKD is driven by a range of metabolic, hemodynamic, inflammatory, and genetic factors, among which oxidative stress plays a central role [6]. Previous studies have revealed that high glucose levels in mouse mesangial cells increase hydrogen peroxide and lipid peroxidation, leading to oxidative stress due to an imbalance between reactive oxygen species (ROS) and antioxidants [7, 8]. Hyperglycemia worsens this stress, causing inflammation and increased ROS production, which can damage cell membranes, proteins, and DNA, leading to cell dysfunction and death [9]. Together, these processes establish a persistent pro‐inflammatory microenvironment within the kidney, underscoring DKD as an inflammation‐driven disease rather than solely a metabolic complication.

Apoptosis‐antagonizing transcription factor (AATF), a regulator with various transcriptional roles, interacts with RNA polymerase II. It is essential for controlling transcription, regulating the cell cycle and apoptosis, responding to DNA damage and stress, binding RNA, and activating inflammatory responses [10, 11, 12]. Previous studies have suggested that AATF may modulate inflammation during sepsis [13]. These findings suggest that AATF may function as a critical molecular node linking cellular stress responses to inflammation‐related signaling pathways.

Transfer RNA‐derived fragments (tRFs) constitute a group of small non‐coding RNAs implicated in the regulation of diverse biological processes [14]. Evidence from high‐throughput sequencing technologies indicates that tRFs are dysregulated in cancer [15]. Additionally, research suggests that tRFs are associated with oxidative stress in radiation‐induced lung injury [16]. Studies further suggest that tRFs may regulate renal immunity, inflammation, and metabolism in the context of renal ischemia‐reperfusion injury [17]. tRFs are crucial for podocyte injury and aberrant proliferation of mesangial cells [14, 18]. tRFs have been shown to participate in the regulation of immune and inflammatory responses by modulating gene expression at the post‐transcriptional level. Therefore, this may play a crucial role in kidney disease. However, the mechanistic impact of tRFs on DKD pathogenesis remains poorly defined.

A previous study found markedly upregulated transforming growth factor β1(TGF‐β1) expression in DKD mice [19, 20]. To investigate the potential functions of tRFs in DKD, we focused on tRF‐15:31‐Arg‐CCT‐3‐1 (tRF‐15:31), which was notably reduced in mouse mesangial cells treated with TGF‐β1 [14]. Although previous studies have implicated specific tRFs in DKD‐related cellular processes, the mechanisms through which individual tRFs regulate oxidative stress and inflammatory responses remain incompletely understood. In particular, the potential relationship between tRF‐15:31 and AATF in DKD has not been characterized. Here, we investigated whether tRF‐15:31 modulates oxidative stress and inflammatory responses in experimental DKD and whether these effects are associated with the regulation of AATF expression. Our study therefore aimed to extend the current understanding of tRF‐mediated regulation in DKD by characterizing the potential relationship between tRF‐15:31 and AATF.

## 2 Materials and Methods

### 2.1 Animal Experiments

Eight‐week‐old male C57BL/6 J (C57) mice (weight, 21–23 g) sourced from Nanjing Medical University were used in the experiments. All animal experiments were performed in accordance with the principles of the Helsinki Declaration and were approved by the Experimental Animal Welfare Ethics Committee of Nanjing Medical University (Approval No. IACUC‐2307056). Sample sizes were determined based on previous studies and pilot experiments. Mice were randomly assigned to four experimental groups (*n* = 5 per group): (1) Control; (2) streptozotocin (STZ); (3) STZ + tRF‐15:31; (4) STZ + tRF‐negative control (NC). Mice were housed in groups of five per cage under controlled conditions (19°C–21°C, 12‐h light/dark cycle) with free access to standard chow and water. Blood glucose levels, urine volume, and urine protein were measured at designated time points. C57 mice, aged 8 weeks, were injected intraperitoneally with STZ at a dosage of 50 mg/kg daily for five consecutive days [21]. The DKD model was successfully established by 12 weeks of age, at which point adeno‐associated virus (AAV) interventions and corresponding NCs were implemented. A single injection of 1 × 10^12^ AAV particles encoding either tRF‐15:31 or tRF‐NC was administered via the tail vein. At 20 weeks of age, mice were anesthetized with sodium barbiturate (100 mg/kg) and euthanized by decapitation for further experimental analysis. Following euthanasia, kidney tissues were immediately harvested for subsequent experimental analyses.

### 2.2 Serum Creatinine (Scr) and 24 h Urine Protein Quantification

The assays were conducted following the manufacturer's instructions (Jiancheng, Nanjing, China). Scr and urine protein levels were measured at designated time points. Blood samples were introduced into 96‐well plates, which included blank, standard, and assay wells, and incubated for 5 min at 37°C. Subsequently, the absorbance was measured at 546 nm, and Scr concentration (µmol/L) was calculated as per the guidelines provided. For assessing urine protein, the prepared samples were dispensed into 96‐well plates, thoroughly mixed, and absorbance was measured at 595 nm. Using the 24 h urine volume of the mice, we determined the 24 h urinary protein content, following the protocol provided by the manufacturer.

### 2.3 Periodic Acid‐Schiff (PAS) Staining

PAS staining was carried out using a commercial kit (Sigma, USA) according to the manufacturer's instructions. Briefly, tissue sections were hydrated, treated with periodic acid for 10 min, incubated in Schiff's reagent for 10 min, and counterstained with hematoxylin for 1 min. Finally, images were gathered by microscopy for analysis.

### 2.4 Immunohistochemistry

Tissue sections underwent a series of preparatory steps, including baking, dewaxing, hydration, and blocking with 5% bovine serum albumin for 2 h. The remaining procedures were carried out according to the manufacturer's guidelines (Maixin, Fuzhou, China). The tissue sections were subsequently incubated with primary antibodies targeting tumor necrosis factor (TNF)‐α and interleukin (IL)‐6. Following incubation with secondary antibodies, the nuclei were stained and sealed. Finally, the microscopic examination was performed, and images were captured for subsequent analysis.

### 2.5 Cell Culture

Mouse glomerular mesangial cells (SV40‐MES 13) were procured from Procell Life Sciences & Technology Co. (Wuhan, China). Cells were grown in Dulbecco's Modified Eagle Medium supplemented with 10% fetal bovine serum and 5.5 mM glucose at 37°C with 5% CO_2_. A high glucose (HG) concentration of 30 mM was established, while a mannitol control (5.5 mM glucose + 24.5 mM mannitol) was used to negate the effects of high osmolarity associated with HG. The experimental method described by Qiao et al. [22] was followed.

### 2.6 Cell Transfection

RiboBio (Guangzhou, China) engineered AATF plasmids to facilitate overexpression. The tRF‐15:31 mimetic, AATF siRNA, serine and arginine‐rich splicing factor 1 (SFRS1), siRNA, and corresponding NCs were procured from RiboBio (Guangzhou, China). Cells were cultured in 6‐ or 12‐well plates 1 day before transfection. When the density reached 50%, transfection was conducted using Lipofectamine 2000 (Invitrogen, Thermo Fisher Scientific Inc., USA) following the manufacturer's instructions. RT‐qPCR and Western blot analyses were used to assess transfection efficiency, and only cells with confirmed transfection were subjected to further experiments.

### 2.7 Reverse‐Transcription qPCR

Total RNA was isolated from kidney tissues or cells using a Total RNA isolation reagent (Invitrogen, CA, USA). For the analysis of tRF‐15:31, 1000 ng of total RNA was reverse‐transcribed using reagents from Vazyme (Nanjing, China). Subsequently, qPCR was conducted to assess the expression of tRF‐15:31, with the expression levels normalized to U6. For mRNA quantification, cDNA synthesis was synthesized using HIScript III RT SuperMix (Vazyme, Nanjing, China), followed by qPCR employing ChamQ Universal SYBR qPCR Master Mix (Vazyme, Nanjing, China). β‐Actin was used as an internal reference for data normalization. The thermocycling conditions were as follows: The reverse transcription product of tRF‐15:31 was incubated at 95°C for 5 min, while the reverse transcription products of other mRNAs were incubated for 30 s at 95°C, followed by maintaining at 95°C for 10 s, at 60°C for 30 s, for 40 cycles. Subsequently, the melting process was conducted and maintained at 60°C for 1 min and at 95°C for 15 s. The data were calculated using the 2^–∆∆Cq^ method [23]. Each primer was provided by GeneRay Biotech (Shanghai, China). The sequences are listed in Table 1.

**Table 1:** Sequences of primers for RT‐qPCR.

Gene	Primer sequence 5′–3′
Mus‐TNF‐α‐ F	CCTTATCTACTCCCAGGTTCTC
Mus‐TNF‐α‐ R	GAGGCTGACTTTCTCCTGGTATG
Mus‐ IL‐6‐ F	CTGCAAGAGACTTCCATCCAG
Mus‐ IL‐6‐ R	AGTGGTATAGACAGGTCTGTTGG
Mus‐AATF‐ F	GAGTGATGATGCCAGGACGGA
Mus‐AATF‐ R	ACTGTCACTTCCCACGGTCTG
Mus‐SFRS1‐ F	CCGTACGCTAGGCTTGGTAG
Mus‐SFRS1‐ R	CGTGAACACCCTTGCCTGTA
Mus‐β‐actin‐ F	CATCCGTAAAGACCTCTATGCCAAC
Mus‐β‐actin ‐ R	ATGGAGCCACCGATCCACA

Abbreviations: AATF, apoptosis‐antagonizing transcription factor; IL‐6, interleukin‐6; RT‐qPCR, reverse‐transcription quantitative polymerase chain reaction; SFRS1, serine and arginine‐rich splicing factor 1; TNF‐α, tumour necrosis factor‐α.

### 2.8 Western Blot

The kidney tissue or mesangial cells were lysed using radioimmunoprecipitation assay (RIPA) lysis buffer supplemented with phenylmethanesulfonyl fluoride (PMSF) protease inhibitor (Beyotime, Shanghai, China) at a ratio of 100:1. The resulting suspension was incubated on ice for 1 h to facilitate lysis. Subsequently, the suspension was centrifuged at 12,000 × g and 4°C for 30 min. Proteins (20 µg/group) were then resolved by SDS‐PAGE and transferred onto a PVDF membrane (Merck Millipore, USA). The membrane was treated with 5% skim milk powder for 1 h, then incubated overnight with the primary antibody at 4°C. After three TBST washes, the membrane was exposed to the secondary antibody at room temperature for 1.5 h. The protein bands were visualized after three washes with TBST. The primary antibodies used in the immunoblot analysis included anti‐ tumor necrosis factor‐alpha (TNF‐α) (1:1000, AF11100, Aifang, Hunan, China), anti‐monocyte chemoattractant protein‐1 (MCP‐1) (1:1000, R09052966, Wanlei, Shenyang, China), anti‐glutathione peroxidase 4 (GPX4) (1:1000, ab125066, Abcam, Cambridge, UK), anti‐AATF (1:1000, 10282‐1‐AP, Proteintech, Wuhan, China), and anti‐SFRS1 (1:5000, 66671‐1‐Ig, Proteintech, Wuhan, China). Horseradish peroxidase‐conjugated goat anti‐rabbit and goat anti‐mouse secondary antibodies (1:10,000) were purchased from Abbkine (Wuhan, China). β‐Tubulin (1:1,000; AF7011; Affinity Biosciences, Changzhou, China) served as the loading control.

### 2.9 Enzyme‐Linked Immunosorbent Assay (ELISA) Analysis

Cell supernatants and mouse serum samples were collected to quantify the concentrations of TNF‐α and IL‐6 using their respective assay kits, following the manufacturer's instructions from Abbkine (Wuhan, China).

### 2.10 Malondialdehyde (MDA) Assay

The MDA levels were quantified using a microassay kit procured from Abbkine (Wuhan, China), adhering strictly to the manufacturer's protocol. Approximately 0.1 g of tissue was accurately weighed, followed by the addition of 1 mL of pre‐cooled extraction buffer. The samples were then homogenized on ice. Subsequently, centrifugation was conducted at 13,000 × g for 10 min at 4°C, and the supernatant was collected for further analysis. For mesangial cells, ultrasonic disruption was used, and the resultant cell lysate was centrifuged at 13,000 g for 10 min at 4°C to eliminate cellular debris. The supernatant was collected, and the reagents were prepared following the kit instructions. Samples were heated at 95°C for 30 min, followed by centrifugation at 10,000 × g for 10 min at room temperature. Absorbance measurements at 532 and 600 nm were conducted using a 96‐well plate. The MDA content was subsequently calculated.

### 2.11 Glutathione (GSH) Assay

Following the protocol provided, GSH levels in mesangial cells and renal tissue were quantified using a GSH detection kit supplied by Abbkine (Wuhan, China). Following cell or tissue lysis, the supernatant was collected via centrifugation, and a standard GSH solution was prepared at a concentration of 200 μg/mL. The GSH content was then determined by measuring the absorbance at 412 nm and referencing a standard calibration curve.

### 2.12 ROS Assay

ROS were quantified utilizing the ROS detection fluorometric assay kit procured from Abbkine (Wuhan, China). The ROS probe (2′,7′‐Dichlorofluorescin diacetate, DCFH‐DA) was prepared in a serum‐free medium to achieve a final concentration of 5 µmol/L. Following the washing of cells with serum‐free medium, the cells were harvested, and the medium was removed prior to the addition of the probe. Subsequently, the cells were incubated at 37°C for 30 min in the absence of light. After incubation, the probes were removed, and the cells were resuspended in phosphate‐buffered saline (PBS). Flow cytometry was then employed to assess ROS levels across different experimental groups.

### 2.13 RNA Immunoprecipitation (RIP)

Protein‐RNA interactions were characterized through RIP analysis, employing the Magna RIP Kit (Millipore, Massachusetts, USA). Cell samples were harvested and thoroughly lysed in RIP lysis buffer in accordance with the manufacturer's protocol. Subsequently, the RIP lysate was incubated with magnetic beads conjugated to a mouse anti‐SFRS1 antibody or with anti‐mouse IgG magnetic beads as a negative control. The RNA associated with the magnetic beads was then extracted and validated using RT‐qPCR.

### 2.14 Co‐Immunoprecipitation (co‐IP) Assays

Following harvesting and washing, the cells were lysed using an IP lysis buffer supplemented with protease inhibitors, and subsequently subjected to centrifugation to obtain the total cell lysate. A/G beads were washed twice with PBST and incubated for 1 h with a negative control IgG and an anti‐SFRS1 antibody. Subsequently, the Protein A/G beads underwent three additional washes with PBST before being incubated overnight at 4°C with the total cell lysate. Immunoprecipitated complexes were eluted by heating in 1 × SDS sample buffer at 100°C for 5 min. Subsequently, Western blot analysis was conducted to assess protein expression levels.

### 2.15 RNA Stability Assay

To measure the stability of AATF mRNA, the mesangial cells were cultured in 12‐well plates and treated with actinomycin D at 5 μg/mL to block transcription. After addition of actinomycin D, cells were collected at various time points (0, 2, 4, 6, and 8 h) and total cellular RNA was isolated. The RNA half‐life was measured using RT‐qPCR.

### 2.16 Statistical Analysis

Results are presented as the mean ± standard deviation, and all statistical analyses were performed using GraphPad Prism (version 8.0.1; GraphPad Software Inc.). Differences between two groups were assessed using an unpaired Student's t‐test, whereas comparisons among multiple groups were conducted by one‐way analysis of variance (ANOVA). *p* < 0.05 was considered statistically significant.

## 3 Results

### 3.1 tRF‐15:31 was Downregulated in DKD Models

As quantitative indicators of renal function, the levels of 24‐h urine protein and blood creatinine were significantly elevated in the STZ group at 20 weeks of age than Control group (Figure 1A). In the STZ group, PAS staining demonstrated significantly increased glycogen content, and immunohistochemical analysis further revealed a marked upregulation of TNF‐α and IL‐6 expression (Figure 1B). These results confirmed the successful establishment of the DKD mice.

**Figure 1:**
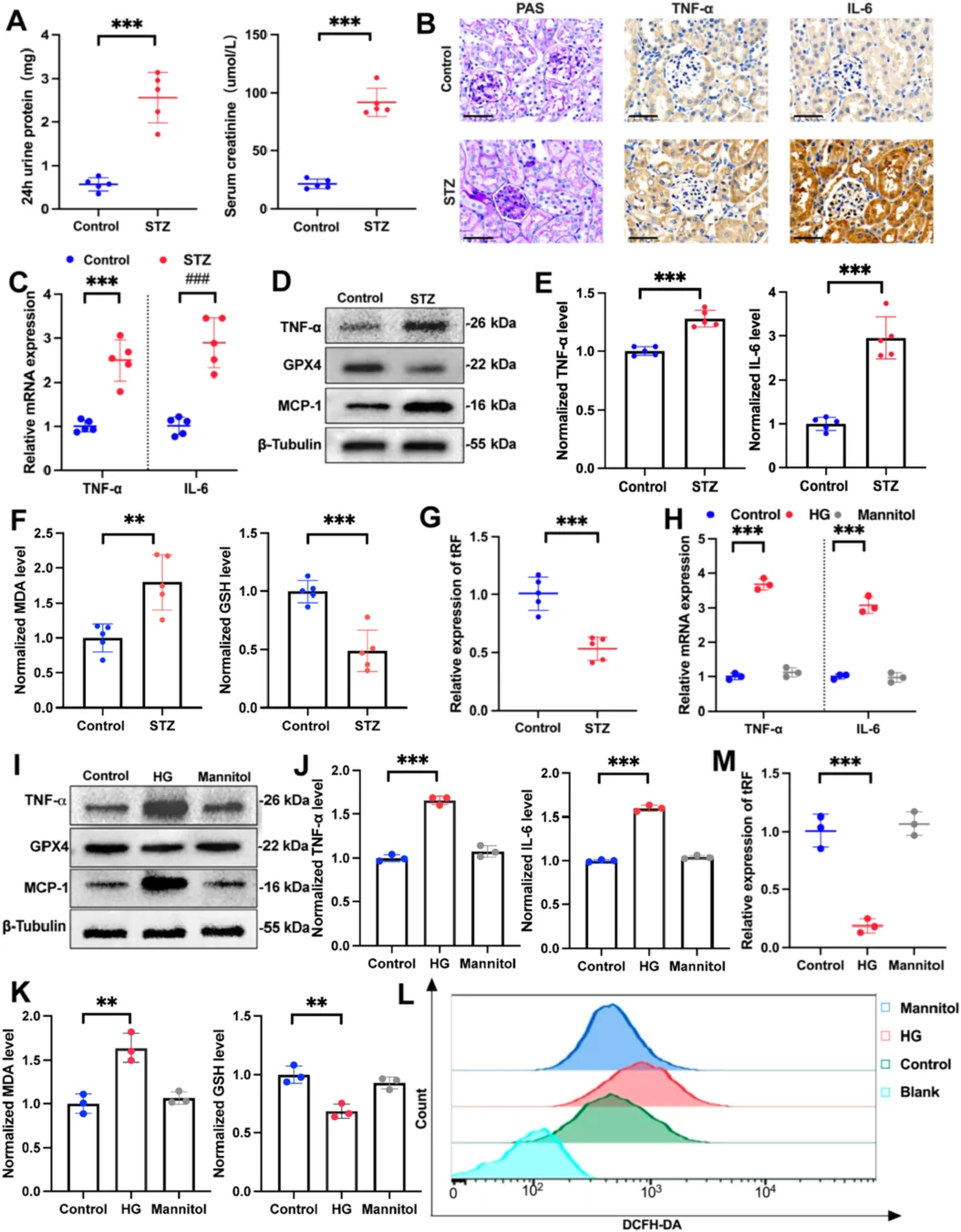
tRF‐15:31 is downregulated in DKD models. (A) The levels of 24‐h urine protein and sCr were measured in mice at 20 weeks of age (*n* = 5). (B) Representative images of PAS‐stained renal cortex glomeruli were obtained, and immunohistochemistry was employed to evaluate the expression of TNF‐α and IL‐6 in renal tissues, with a scale bar of 50 μm. (C, E) In kidney tissues of the mice, the expression of TNF‐α and IL‐6 was quantified using RT‐qPCR, while their serum levels were assessed using enzyme‐linked immunosorbent assay (ELISA) (*n* = 5). (D) Western blot analysis was conducted to detect the protein expression levels of TNF‐α, GPX4, and MCP‐1. (F) The expression levels of MDA and GSH were also measured in the kidney tissues of the mice (*n* = 5). (G) The expression levels of tRF‐15:31 in each experimental group were validated using RT‐qPCR (*n* = 5). (H, J) The mRNA levels of TNF‐α and IL‐6 in HG‐treated mesangial cells were also quantified via RT‐qPCR, while their corresponding protein levels were determined using ELISA (*n* = 3). (I) Western blot analysis was employed to detect the expression of TNF‐α, GPX4, and MCP‐1 in mesangial cells exposed to HG conditions. (K, L) Cellular levels of MDA and GSH were measured using specific MDA and GSH assays and ROS levels were evaluated through flow cytometry (*n* = 3). (M) The expression of tRF‐15:31 in HG‐induced mesangial cells was quantified using RT‐qPCR, with U6 serving as the normalization control (*n* = 3). The results are presented as the mean ± standard deviation (SD). **p < 0.01, ***p < 0.001, ###*p* < 0.001 versus the indicated group.

To evaluate oxidative stress and inflammation, we analyzed the kidney tissue levels of MDA, GSH, and serum cytokines (TNF‐α and IL‐6). In the STZ group, MDA, TNF‐α, and IL‐6 levels were significantly higher compared to the Control group, whereas GSH levels were lower (Figure 1C,E,F). Subsequent Western blot analysis indicated significantly elevated levels of TNF‐α and MCP‐1, alongside a reduced expression of GPX4 in kidney tissues of the STZ group compared to the Control group (Figure 1D). To investigate the role of tRF‐15:31 in DKD, we measured its expression in kidney tissues of DKD mice using RT‐qPCR. We confirmed that tRF‐15:31 expression was downregulated in the STZ group via RT‐qPCR (Figure 1G). *In vitro*, the expression levels of TNF‐α and IL‐6 were significantly elevated compared to the control group, as determined by RT‐qPCR (Figure 1H). Western blot analysis further revealed that TNF‐α and MCP‐1 levels were significantly higher, while GPX4 levels were lower in the HG group (Figure 1I). Furthermore, MDA, ROS, and inflammatory markers (TNF‐α and IL‐6) increased, whereas the oxidative stress marker GSH decreased in the HG group (Figure 1J–L). RT‐qPCR analysis confirmed low expression levels of tRF‐15:31 in the HG group (Figure 1M). In contrast, the mannitol group exhibited non‐significant changes (Figure 1H,J–L), indicating that high osmotic pressure did not affect oxidative stress and inflammation. The results implied that tRF‐15:31 might be important for oxidative stress and inflammation in DKD.

### 3.2 tRF‐15:31 Alleviated Oxidative Stress and Inflammation In Vivo

To further explore the relationship between tRF‐15:31 and oxidative stress and inflammation in DKD mice, we conducted follow‐up experiments. The DKD model was successfully established in C57BL/6 J mice at 12 weeks, as revealed by blood glucose, 24‐h urine volume, and urinary protein measurements (Figure S1). To investigate the effects of tRF‐15:31 overexpression, we administered an AAV via the tail vein, using AAV‐NC as a control. At 20 weeks of age, the successful overexpression of tRF‐15:31 was confirmed by elevated levels of tRF‐15:31 mRNA (Figure 2A). Subsequently, we assessed inflammation and oxidative stress in the mice with DKD. Blood glucose, creatinine, and 24‐h urinary protein levels were measured in 20‐week‐old mice. The findings indicated that the STZ + tRF‐15:31 group exhibited lower creatinine and 24‐h urinary protein levels compared to the STZ + tRF‐NC group, while non‐significant differences were observed in blood glucose levels (Figures 2B and S2). In the STZ + tRF‐15:31 group, PAS staining demonstrated significantly reduced glycogen content, and immunohistochemical analysis further revealed that the expression levels of TNF‐α and IL‐6 were markedly lower compared to the STZ + tRF‐NC group (Figure 2C). Compared to the STZ + tRF‐NC group, the oxidative stress and inflammation were reduced in the STZ + tRF‐15:31 group, as evidenced by decreased mRNA levels of TNF‐α, IL‐6, and reduced protein levels of TNF‐α and MCP‐1, and increased protein level of GPX4 (Figure 2D,E). Moreover, serum levels of IL‐6, TNF‐α, and MDA levels were reduced, while GSH levels were higher in the STZ + tRF‐15:31 group (Figure 2F,G). Collectively, these findings suggest that oxidative stress and inflammatory damage were attenuated in the STZ + tRF‐15:31 group relative to the STZ + tRF‐NC group.

**Figure 2:**
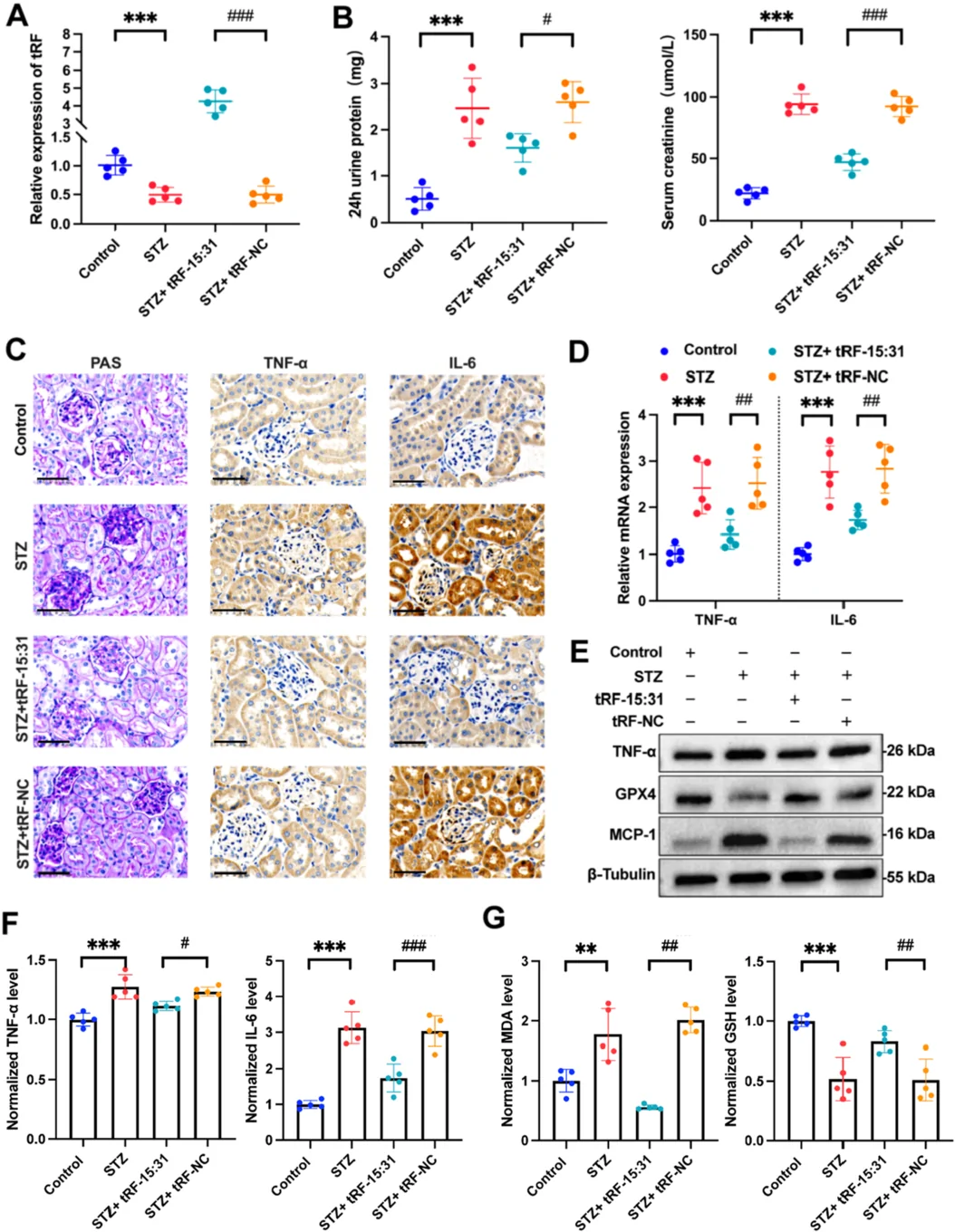
tRF‐15:31 alleviates oxidative stress and inflammation in vivo. (A) The overexpression of tRF‐15:31 in vivo was confirmed using RT‐qPCR, with U6 serving as the normalization control (*n* = 5). (B) The 24‐h urine protein and sCr levels were measured in each group of mice (*n* = 5). (C) In DKD mice, PAS staining images revealed that tRF‐15:31 administration led to improvements in glomerular morphology and immunohistochemical analysis indicated significant reductions in inflammatory markers, including TNF‐α and IL‐6. The scale bar represents 50 μm. (D, F) The expression levels of typical inflammatory factors, such as TNF‐α and IL‐6, were quantified using RT‐qPCR and ELISA assays across all groups of mice (*n* = 5). (E) Western blot analysis was conducted to assess the levels of TNF‐α, MCP‐1, and GPX4 in the kidney tissues. (G) Additionally, MDA and GSH were detected in the kidney tissues of all mouse groups (*n* = 5). The results are presented as the mean ± standard deviation (SD). **p < 0.01, ***p < 0.001, ^#^ *p* < 0.05, ^##^ *p* < 0.01, ^###^ *p* < 0.001 versus the indicated group.

### 3.3 tRF‐15:31 Inhibited Oxidative Stress and Inflammation In Vitro

To elucidate the regulatory effects of tRF‐15:31 on oxidative stress and inflammation, a tRF‐15:31 mimetic was overexpressed in HG‐treated mesangial cells, and its overexpression was successfully verified using RT‐qPCR (Figure 3A). Subsequently, the HG+tRF‐15:31 group revealed a significant decrease in mRNA levels of TNF‐α and IL‐6 according to RT‐qPCR, and ELISA results demonstrated decreased levels of inflammatory cytokines (TNF‐α and IL‐6) in the cell supernatant (Figure 3B,D). The HG+tRF‐15:31 group experienced a reduction in MDA and ROS levels, along with a rise in GSH level, while TNF‐α and MCP‐1 proteins decreased and GPX4 proteins went up (Figure 3C,E,F). Altogether, these data indicated that tRF‐15:31 may protect against DKD by inhibiting mesangial cell oxidative stress and inflammation.

**Figure 3:**
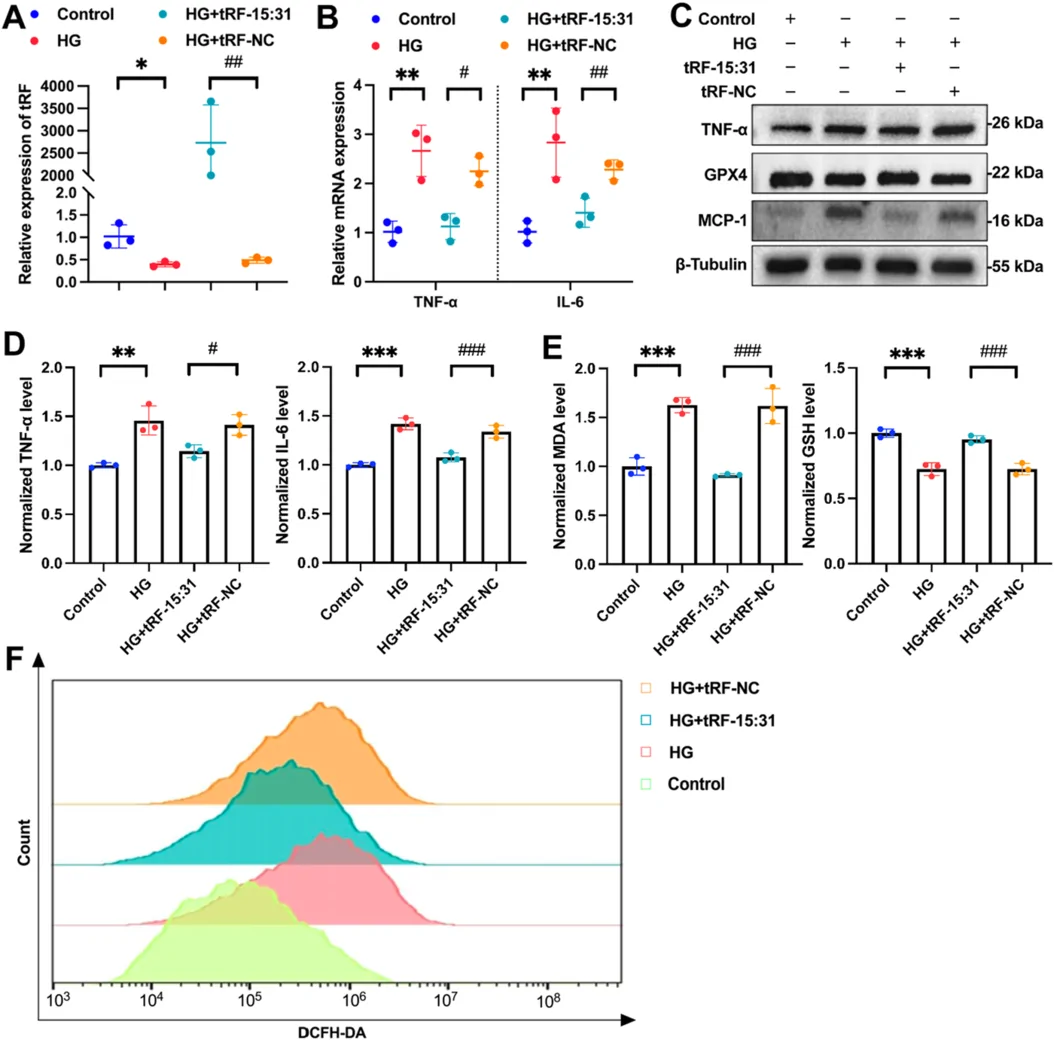
tRF‐15:31 inhibits oxidative stress and inflammation in vitro. (A, B, D). The study involved the overexpression of tRF‐15:31 in mesangial cells treated with HG, followed by the assessment of TNF‐α and IL‐6 expression levels using RT‐qPCR and ELISA (*n* = 3). (C) Additionally, the expression of TNF‐α, GPX4, and MCP‐1 in the tRF‐15:31 overexpression group was analyzed using Western blot. (E,F) Furthermore, the levels of MDA and GSH were quantified in each experimental group to evaluate oxidative stress and ROS levels were measured via flow cytometry (*n* = 3). The results are presented as the mean ± standard deviation (SD). *p < 0.05, **p < 0.01, ***p < 0.001, ^#^ *p* < 0.05, ^##^ *p* < 0.01, ^###^ *p* < 0.001 versus the indicated group.

### 3.4 tRF‐15:31 Regulated Oxidative Stress and Inflammation by Targeting AATF

Next, we investigated how tRF‐15:31 mitigated oxidative stress and inflammation. The results indicated that AATF mRNA and protein expression were significantly reduced in the STZ+ tRF‐15:31 group compared to the STZ+ tRF‐NC group, as determined by RT‐qPCR and Western blot analyses (Figure 4A,B). *In vitro* experiments involved tRF‐15:31 overexpression in HG‐induced mesangial cells, followed by evaluation of AATF expression using RT‐qPCR and Western blot. The findings demonstrated a significant decrease in AATF expression in the HG+ tRF‐15:31 mimetic group compared to the HG+tRF‐NC group (Figure 4C,D). Several studies have identified an association with AATF in oxidative stress and inflammation [13, 24, 25]. Moreover, following the establishment of a connection between AATF and oxidative stress and inflammation, we explored whether AATF overexpression could counteract the protective effects of tRF‐15:31 against oxidative stress and inflammation in mesangial cells. Our study found that co‐transfection of tRF‐15:31 mimetic and AATF plasmids significantly mitigated the inhibitory effects of tRF‐15:31 on oxidative stress and inflammation. This was demonstrated by notable changes in the levels of TNF‐α, IL‐6, MCP‐1, and GPX4 (Figure 4E,F). These findings suggest that tRF‐15:31 may attenuate oxidative stress and inflammation in DKD through modulating AATF.

**Figure 4:**
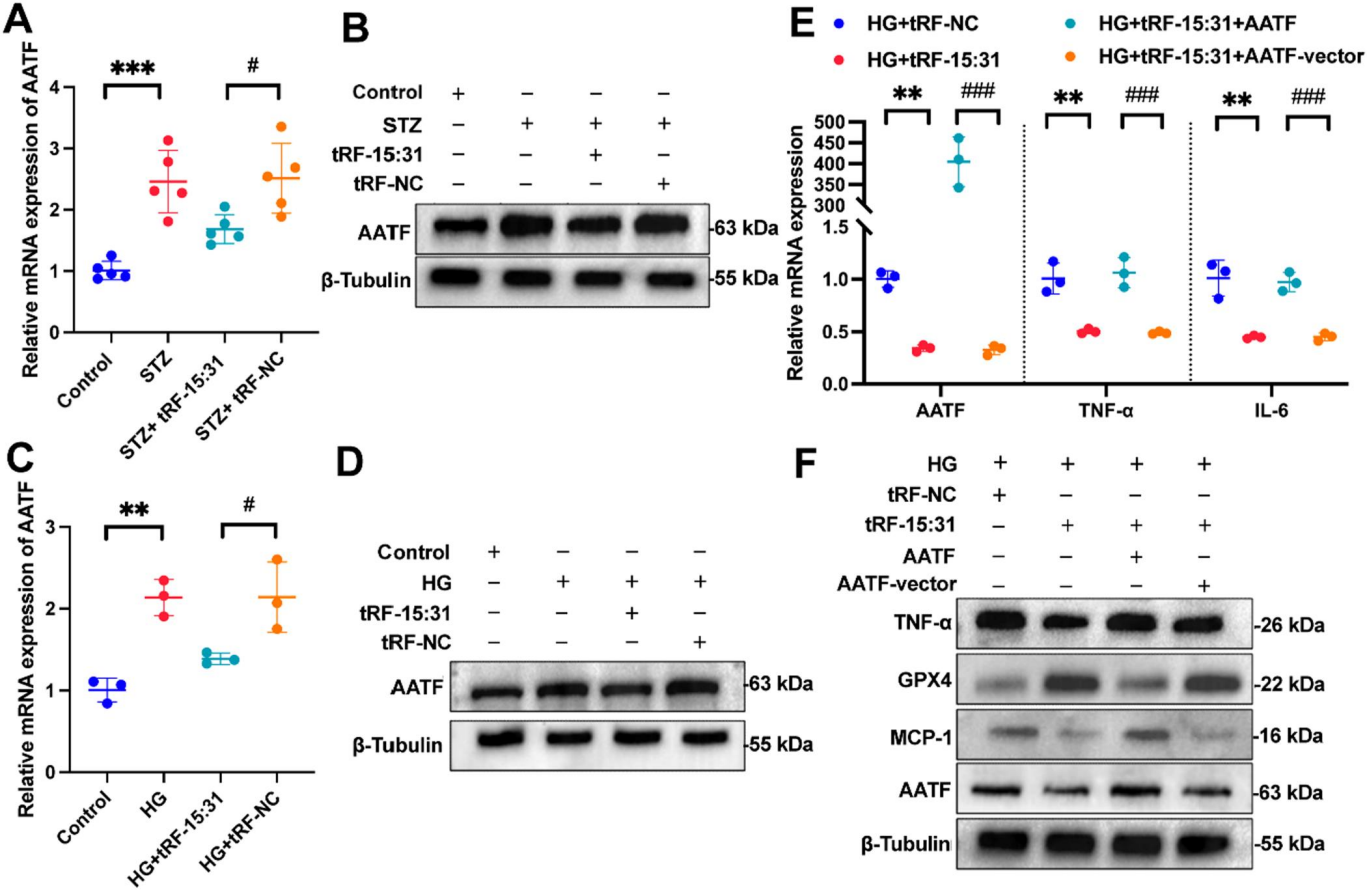
tRF‐15:31 regulates oxidative stress and inflammation by targeting AATF. (A, B)The expression levels of AATF in various groups of mice were confirmed through RT‐qPCR and Western blot analysis (*n* = 5). (C, D) Following the overexpression of tRF‐15:31 in high‐glucose mesangial cells, AATF expression was assessed using RT‐qPCR and Western blot (*n* = 3). (E, F) Additionally, RT‐qPCR and Western blot were employed to evaluate the expression levels of AATF, as well as oxidative stress and inflammation‐related markers, subsequent to the cotransfection of tRF‐15:31 mimetic and AATF plasmids in high‐glucose mesangial cells (*n* = 3). The results are presented as the mean ± standard deviation (SD). Statistical significance is denoted as follows: **p < 0.01, ***p < 0.001, and ^#^ *p* < 0.05 compared to the specified group.

### 3.5 Knockdown of AATF Alleviated Oxidative Stress and Inflammation In Vitro

To investigate the role of AATF in oxidative stress and inflammation, we investigated its expression in DKD models *in vivo* and *in vitro*. Subsequently, our study identified a significant upregulation of AATF in the STZ group within mouse kidney tissue, as determined by RT‐qPCR (Figure 5A) and Western blot analysis (Figure 5B). *In vitro* experiments revealed an increase in AATF mRNA and protein expression levels in the HG group, as measured by RT‐qPCR and Western blot, respectively (Figure 5C,D). This prompted us to investigate whether the inhibition of AATF could influence oxidative stress and inflammation in the HG group. The efficacy of AATF knockdown in the HG + AATF siRNA group was confirmed through RT‐qPCR and Western blot analyses (Figure 5E,F).

**Figure 5:**
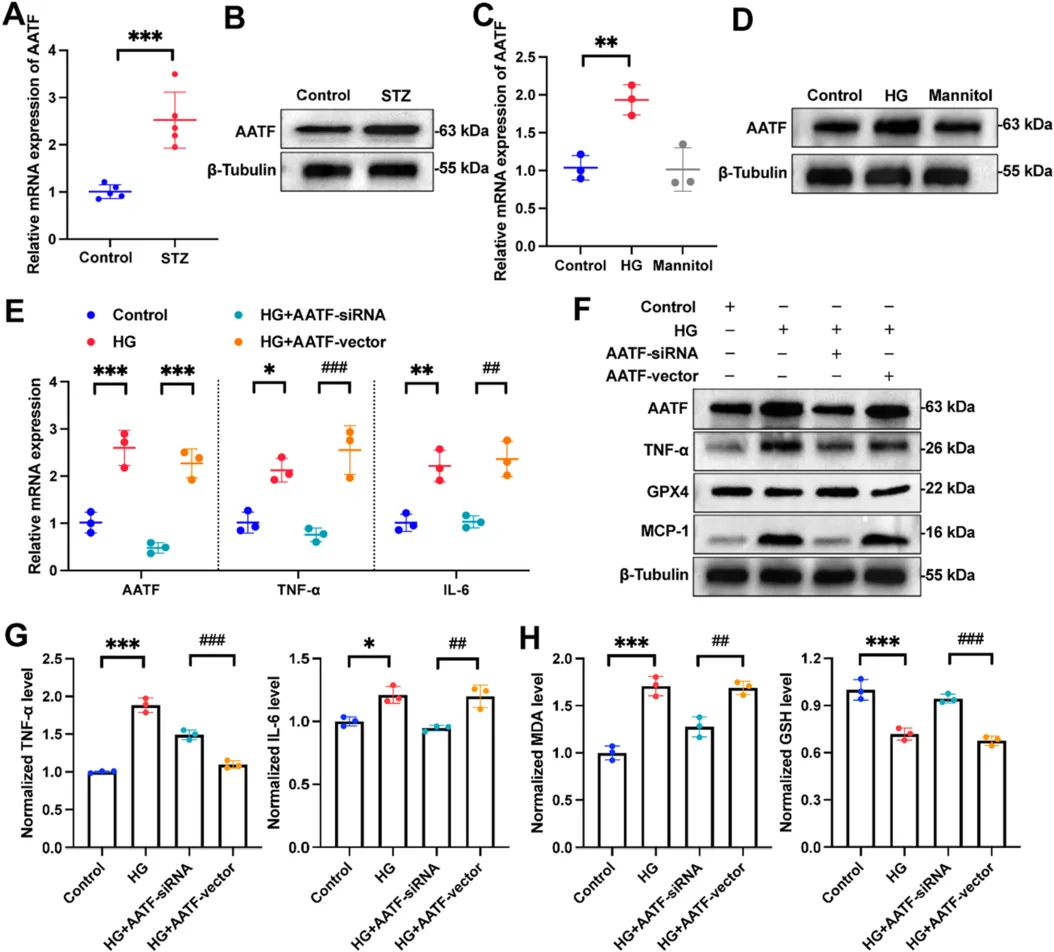
Knockdown of AATF alleviates oxidative stress and inflammation in vitro. (A, B) The expression levels of AATF in DKD mice were quantified using RT‐qPCR and Western blot analysis (*n* = 5). (C, D) RT‐qPCR and Western blot were employed to confirm AATF expression in mesangial cells under high glucose conditions (*n* = 3). (E, F) The knockdown of AATF in these cells was validated through RT‐qPCR and Western blot analysis (*n* = 3). (G, H) We tested the levels of TNF‐α, IL‐6, MDA, and GSH by corresponding kits (*n* = 3). The data are expressed as the mean ± SD. *p < 0.05, **p < 0.01, ***p < 0.001, ^##^ *p* < 0.01, ^###^ *p* < 0.001 versus the indicated group.

Furthermore, indicators related to oxidative stress and inflammatory responses were assessed. Compared with the HG + AATF‐vector group, the levels of TNF‐α and IL‐6 mRNA were reduced as determined by RT‐qPCR, and protein expressions of TNF‐α and MCP‐1 were lowered according to Western blot, while GPX4 protein expression increased in the HG + AATF‐siRNA group (Figure 5E,F). The concentrations of MDA, TNF‐α, and IL‐6 were significantly elevated in the HG + AATF‐vector group compared to the HG + AATF‐siRNA group, whereas the GSH levels exhibited a declining trend (Figure 5G). Our findings demonstrate that AATF knockdown via siRNA effectively attenuated oxidative stress and inflammation in HG‐treated mesangial cells.

### 3.6 tRF‐15:31 Reduced the AATF mRNA Stability Through SFRS1

To explore how tRF‐15:31 controls AATF expression, we performed bioinformatics analysis using the RBP prediction website: SFRS1 might be the shared RBP for tRF‐15:31 and AATF, with a potential binding site of “UGGA” (Figure S3). Preliminary evidence supporting the interaction between tRF‐15:31 and SFRS1 was obtained through RIP assays (Figure 6A). Besides, co‐IP experiments confirmed the binding of AATF to SFRS1 (Figure 6D). To investigate the potential relationship between SFRS1 and AATF mRNA stability, we performed siRNA‐mediated knockdown of SFRS1 in mesangial cells. The efficiency of this interference was confirmed via RT‐qPCR and Western blot (Figure 6B,E). Compared to the SFRS1‐vector group, RT‐qPCR analysis indicated a reduction in AATF mRNA stability following SFRS1 knockdown (Figure 6C). Furthermore, the stability of AATF mRNA in mesangial cells was assessed after the tRF‐15:31 overexpression. The analysis of RT‐qPCR data indicated reduced stability of AATF mRNA in the tRF‐15:31 group compared to the tRF‐NC group (Figure 6F). These results suggest that the association of tRF‐15:31 with SFRS1 is accompanied by reduced AATF mRNA stability and may contribute to the attenuation of oxidative stress and inflammation.

**Figure 6:**
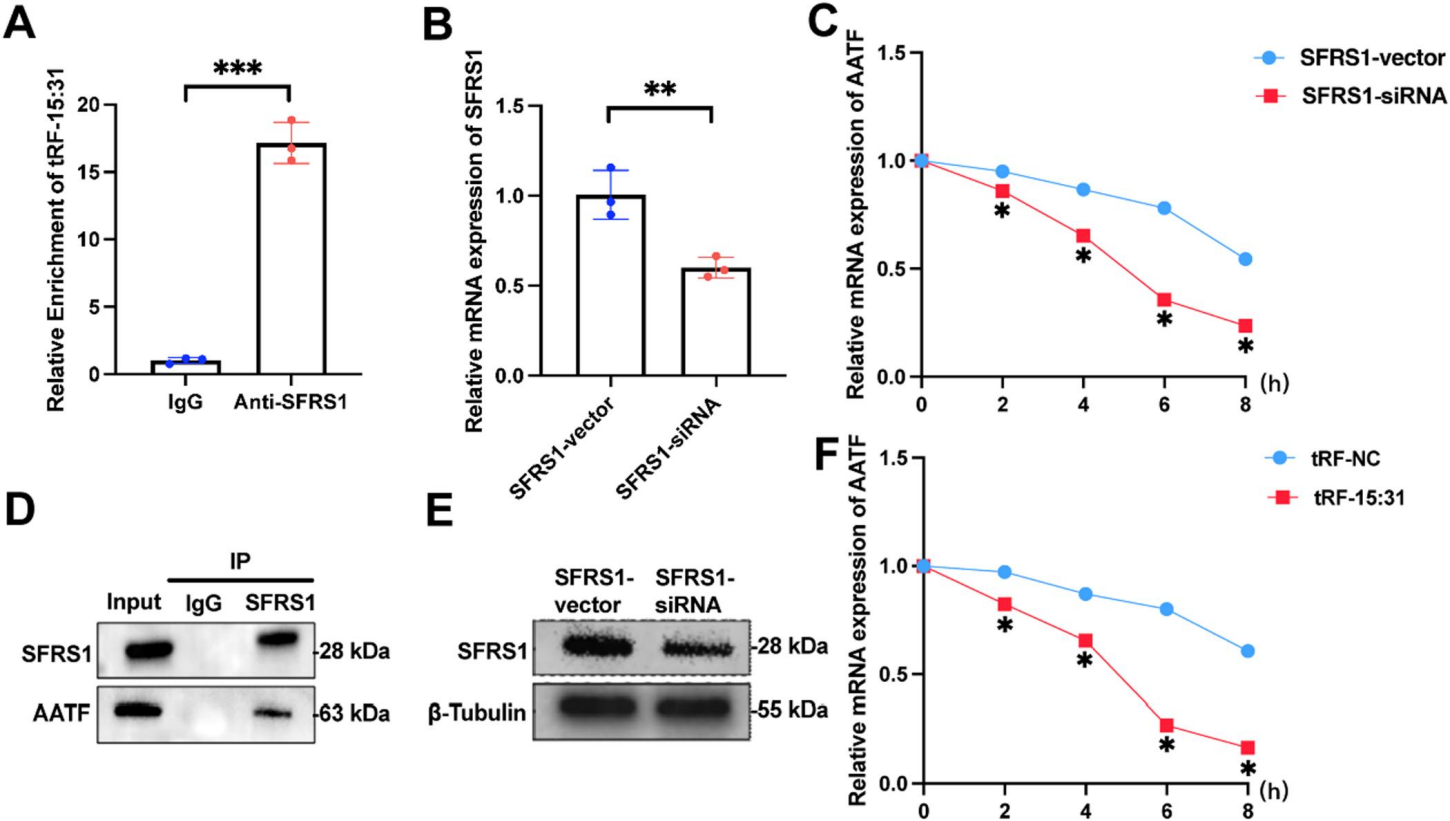
tRF‐15:31 reduced the stability of AATF mRNA through SFRS1. (A) The enrichment of SFRS1 was quantified using RIP‐qPCR. (B, E) The efficiency of SFRS1 interference was assessed via RT‐qPCR and Western blot. (C) The expression levels of AATF mRNA were analyzed by RT‐qPCR subsequent to the knockdown of SFRS1. (D) The interaction between SFRS1 and AATF was confirmed through Co‐immunoprecipitation followed by Western blot analysis. (F) The expression levels of AATF mRNA were measured using RT‐qPCR following the overexpression of tRF‐15:31. *n* = 3, *p < 0.05,**p < 0.01, ***p < 0.001 versus the indicated group.

## 4 Discussion

DKD is the primary cause of end‐stage renal disease globally, and its progression is poorly controlled even with strict blood pressure and glucose management [26]. Understanding its pathogenesis is crucial for finding new biomarkers and treatments. Oxidative stress and inflammation are significant factors in DKD's development [27]. tRFs, a new type of small non‐coding RNAs, are important in various biological and physiological processes [28]. Our findings suggest that tRF‐15:31 is associated with SFRS1 and reduced AATF mRNA stability, accompanied by attenuation of oxidative stress and inflammation in mesangial cells. These findings support further investigation of the biological significance of tRF‐15:31 in DKD.

tRFs, produced at tRNA sites by specific nucleases, are increasingly associated with diseases such as neuronal degeneration, cancer, infections, and kidney issues [29]. tRF3‐IleAAT suppresses ferroptosis through downregulation of ZNF281, thereby attenuating extracellular matrix (ECM) synthesis in mesangial cells and slowing the progression of DKD [22]. Together with previous studies implicating tRFs in mesangial cell proliferation and podocyte biology [14, 18], these findings support the involvement of specific tRFs in DKD‐related cellular processes. The present findings therefore extend this existing framework by providing additional evidence for the involvement of tRF‐15:31 in oxidative stress and inflammatory responses in DKD. However, the mechanisms through which individual tRFs regulate oxidative stress and inflammation remain incompletely understood. Consequently, the initiation of oxidative stress and inflammation is a critical entry point in this pathological process. MDA, GSH, and GPX4 are vital indicators that reflect the level of oxidative stress [30, 31].

Additionally, TNF‐α, IL‐6, and MCP‐1 are pivotal in mediating the inflammatory response [32]. In the present study, tRF‐15:31 was found to be downregulated in both STZ‐induced DKD mice and HG‐treated mesangial cells, accompanied by increased oxidative stress and inflammatory responses. Overexpression of tRF‐15:31 improved renal functional and histopathological parameters in DKD mice and attenuated oxidative stress and inflammation, with similar effects observed in vitro. Collectively, these findings indicate that tRF‐15:31 may contribute to the attenuation of DKD‐associated injury by modulating oxidative stress and inflammatory responses in mesangial cells.

We further investigated the molecular mechanisms through which tRF‐15:31 mediates its antioxidative and anti‐inflammatory effects. Notably, the expression levels of AATF mRNA and protein were significantly reduced in the STZ + tRF‐15:31 group compared to the STZ + tRF‐NC group, raising our interest. Studies revealed that AATF is associated with various diseases, enhancing tumor progression in a kirsten rat sarcoma viral oncogene (KRAS)‐driven lung cancer model by impeding p53‐mediated apoptosis and playing a key role in immune regulation within the mitochondrial pathway of apoptosis [33, 34]. Although AATF has been implicated in oxidative stress and inflammatory regulation, its role in DKD remains insufficiently characterized. Our existing data suggested that tRF‐15:31 overexpression can suppress the protein level of AATF in mesangial cells treated with high glucose. To further clarify the role of AATF in modulating oxidative stress and inflammatory responses linked to DKD, an *in vitro* study was performed, in which AATF expression was knocked down in HG‐treated mesangial cells. This intervention resulted in a marked reduction in oxidative stress and inflammatory responses, supporting the involvement of AATF in regulating these processes. Through the co‐transfection of tRF‐15:31 mimic and AATF plasmids, the inhibitory effects of tRF‐15:31 on oxidative stress and inflammation were significantly reversed. These findings suggested that tRF‐15:31 may contribute to the attenuation of oxidative stress and inflammation in DKD mesangial cells by downregulating AATF expression.

tRFs exert biological functions through diverse mechanisms, such as interacting with RNA‐binding proteins to regulate the stability of target gene mRNA [35, 36]. We found that SFRS1 can be associated with tRF‐15:31 and AATF. SFRS1, a member of the serine/arginine‐rich protein family, is essential for regulating mRNA stability. This regulatory mechanism is analogous to that of other well‐characterized mRNA stability regulators, such as ADAR2 and Act1, which modulate mRNA stability and translational efficiency through mRNA binding mechanisms [37, 38]. In the present study, our findings suggest that the interaction of tRF‐15:31 with SFRS1 is associated with reduced AATF mRNA stability and expression. Taken together, these findings provide additional mechanistic evidence linking tRF‐15:31 to AATF regulation and suggest that the tRF‐15:31‐SFRS1‐AATF regulatory relationship may contribute to oxidative stress and inflammatory responses in experimental DKD.

DKD is a multifactorial disease driven by interconnected metabolic, hemodynamic, oxidative, and inflammatory processes [39]. Persistent hyperglycemia and associated metabolic disturbances can promote ROS generation and inflammatory signaling through multiple interconnected pathways, highlighting the complex interplay between metabolic, oxidative, and inflammatory processes [40]. Therefore, the tRF‐15:31‐SFRS1‐AATF regulatory relationship identified in the present study should be considered as one component of this broader regulatory network rather than as an isolated pathway that fully accounts for DKD pathogenesis. Our findings suggest that this regulatory relationship may contribute to the modulation of oxidative stress and inflammatory responses in mesangial cells, potentially linking tRF‐mediated post‐transcriptional regulation with established pathogenic processes in DKD. Further studies are required to determine how this regulatory relationship interacts with other metabolic, inflammatory, and oxidative pathways during DKD progression. Systems‐level and computational approaches may provide a useful framework for further investigating these interactions. Network‐based modeling has been used to integrate interconnected metabolic, oxidative, and inflammatory pathways under hyperglycemic conditions and to identify potentially influential components within complex biological networks [40]. Future integration of such approaches with experimental data may help position the tRF‐15:31‐SFRS1‐AATF regulatory relationship within the broader DKD regulatory network and identify potential interactions with other disease‐associated pathways.

Several limitations of this study should be acknowledged. First, the present findings were derived exclusively from an STZ‐induced mouse model and cultured mouse mesangial cells, without validation in human DKD samples. Therefore, whether the altered expression and regulatory effects of tRF‐15:31 observed in these experimental models are also present in patients with DKD remains to be determined. Future studies incorporating human samples are needed to evaluate the clinical relevance of tRF‐15:31 and the tRF‐15:31‐SFRS1‐AATF regulatory relationship.

## 5 Conclusions

This study investigated the role and mechanism of tRF‐15:31 in a DKD animal model and mesangial cells exposed to high glucose conditions. Our findings suggest that tRF‐15:31 interacts with SFRS1 to reduce AATF mRNA stability, thereby reducing its expression and alleviating oxidative stress and inflammation in mesangial cells, providing additional mechanistic evidence for the involvement of tRF‐mediated AATF regulation in experimental DKD. These findings expand our understanding of the regulatory mechanisms underlying oxidative stress and inflammation in DKD; however, their relevance to human DKD and potential therapeutic implications require further investigation.

## AI Usage Statement

AI‐assisted tools were used during manuscript preparation solely for language polishing and grammar correction. All AI‐assisted revisions were critically reviewed and verified by the authors. The authors take full responsibility for the content of the final manuscript. All scientific interpretations, data analyses, and conclusions were made solely by the authors.

## Author Contributions

**Beibei Ning:** visualization, validation, writing – original draft, methodology, investigation, formal analysis; data curation. **Chan Huang:** investigation, formal analysis, validation, data curation. **Xuewei Liu:** investigation, data curation. **Yunyang Qiao:** investigation, formal analysis. **Jialing Ji:** investigation, formal analysis. **Huimin Shi:** methodology, visualization. **Gaoting Qu:** investigation, formal analysis. **Yajie Zhao:** methodology, resources. **Weihua Gan:** methodology, visualization, resources. **Aiqing Zhang:** writing – review and editing, visualization, resources, methodology, investigation, conceptualization.

## Ethics Statement

All experimental procedures were conducted in compliance with the Helsinki Declaration and received approval from the Experimental Animal Welfare Ethics Committee of Nanjing Medical University (Approval No. IACUC‐2307056).

## Conflicts of Interest

The authors declare no conflicts of interest.

## Supporting information


Supporting File 1

Supporting File 2

## Data Availability

The data that support the findings of this study are available from the corresponding author upon reasonable request.
