# Supplementary material for: tRF‐15:31‐Arg‐CCT‐3‐1 Improves Diabetic Kidney Disease by Reducing AATF and Alleviating Mesangial Cell Oxidative Stress and Inflammation
Source: Immun Inflamm Dis. 2026 Oct 19;14(10):e70561. doi: 10.1002/iid3.70561 (PMC13678860; doi:10.1002/iid3.70561)
Supplement: Supplementary file 1 [file IID3-14-e70561-s002.pdf]

NOTE: Please save this file locally before filling in the table, DO NOT work on the file within your internet browser as changes will not be saved. Adobe Acrobat Reader (available free here) is recommended for completion.

The ARRIVE guidelines 2.0: author checklist

The ARRIVE Essential 10

These items are the basic minimum to include in a manuscript. Without this information, readers and reviewers cannot assess the reliability of the findings.

Item		Recommendation	Section/line number, or reason for not reporting
**Study design**	1	For each experiment, provide brief details of study design including:	
		a. The groups being compared, including control groups. If no control group has been used, the rationale should be stated.	Sec. 2.1 describes four groups: Control, STZ, STZ + tRF-15:31, and STZ + tRF-negative control
		b. The experimental unit (e.g. a single animal, litter, or cage of animals).	Sec. 2.1 Mice assigned to the same experimental group were housed together. The individual mouse was the experimental unit.
**Sample size**	2	a. Specify the exact number of experimental units allocated to each group, and the total number in each experiment. Also indicate the total number of animals used.	Section 2.1: 20 mice in total, with 5 mice allocated to each of the four experimental groups.
		b. Explain how the sample size was decided. Provide details of any *a priori* sample size calculation, if done.	Section 2.1: The sample size was based on previous studies and pilot experiments. No formal a priori sample size calculation was performed.
**Inclusion and exclusion criteria**	3	a. Describe any criteria used for including and excluding animals (or experimental units) during the experiment, and data points during the analysis. Specify if these criteria were established *a priori*. If no criteria were set, state this explicitly.	Section 2.1 The only prespecified exclusion criterion for terminal sample analyses was death before the scheduled experimental endpoint.
		b. For each experimental group, report any animals, experimental units or data points not included in the analysis and explain why. If there were no exclusions, state so.	Section 2.1 No mice died before the scheduled experimental endpoint, and no animals were excluded from any experimental group.No data points were
		c. For each analysis, report the exact value of *n* in each experimental group.	For each in vivo outcome analysed, the sample size was five individual mice per experimental group (n = 5 per group).
**Randomisation**	4	a. State whether randomisation was used to allocate experimental units to control and treatment groups. If done, provide the method used to generate the randomisation sequence.	Section 2.1 Mice were individually numbered and randomly allocated to four experimental groups using a computer-generated randomization sequence (n = 5 mice per group).
		b. Describe the strategy used to minimise potential confounders such as the order of treatments and measurements, or animal/cage location. If confounders were not controlled, state this explicitly.	Section 2.1 To minimise potential confounding, cage positions and the order of treatment administration and measurements were randomised. Each experimental procedure was performed by the same operator across all groups. Treatment
**Blinding**	5	Describe who was aware of the group allocation at the different stages of the experiment (during the allocation, the conduct of the experiment, the outcome assessment, and the data analysis).	Section 2.1The investigators responsible for group allocation and treatment administration were aware of the group assignments. All other personnel, including those involved in sample processing, data collection, outcome assessment, and statistical analysis, were blinded to treatment
**Outcome measures**	6	a. Clearly define all outcome measures assessed (e.g. cell death, molecular markers, or behavioural changes).	Sections 2.1–2.4 and 2.7–2.11; figure legends for Figures 1, 2, 4 and 5.
		b. For hypothesis-testing studies, specify the primary outcome measure, i.e. the outcome measure that was used to determine the sample size.	No single primary outcome measure was prespecified. The sample size of five mice per group was determined based on previous studies and pilot experiments.
**Statistical methods**	7	a. Provide details of the statistical methods used for each analysis, including software used.	Section 2.16 Data were expressed as the mean ± standard deviation. Statistical analyses were performed using GraphPad Prism version 8.0.1. Comparisons between two groups were conducted using an
		b. Describe any methods used to assess whether the data met the assumptions of the statistical approach, and what was done if the assumptions were not met.	Section 2.16 For the animal experiments, homogeneity of variance was assessed. No formal normality tests were performed. With only five mice per group, such tests have
**Experimental animals**	8	a. Provide species-appropriate details of the animals used, including species, strain and substrain, sex, age or developmental stage, and, if relevant, weight.	Sec. 2.1 identifies male C57BL/6J mice, eight weeks old at experimental use, weighing 21-23 g.
		b. Provide further relevant information on the provenance of animals, health/immune status, genetic modification status, genotype, and any previous procedures.	Section 2.1 Healthy, specific pathogen-free (SPF), non-genetically modified C57BL/6J mice were obtained from Nanjing Medical University. The mice had not undergone any
**Experimental procedures**	9	For each experimental group, including controls, describe the procedures in enough detail to allow others to replicate them, including:	
		a. What was done, how it was done and what was used.	Section 2.1-2.4,2.7-2.11 Mice were assigned to four groups (n = 5 per group): Control, STZ, STZ + tRF-15:31, and STZ + tRF-negative control (NC). Mice in the three
		b. When and how often.	Section 2.1 Mice were obtained at six weeks of age and acclimatised for two weeks before experimental procedures began.
		c. Where (including detail of any acclimatisation periods).	Section 2.1 Animal experiments were conducted at the Animal Experiment Center of Nanjing Medical University. Mice were
		d. Why (provide rationale for procedures).	Section1, 2.1-2.4, 2.7-2.11 STZ was used to induce diabetes and associated kidney injury to investigate tRF-15:31 in
**Results**	10	For each experiment conducted, including independent replications, report:	
		a. Summary/descriptive statistics for each experimental group, with a measure of variability where applicable (e.g. mean and SD, or median and range).	Section Section 2.16 and the corresponding figure legends. Quantitative results for the experimental groups are presented as the mean ± standard deviation (SD) in the figures, as specified in Section 2.16 and the
		b. If applicable, the effect size with a confidence interval.	Section Section 2.16 and Figures. Quantitative results are presented as means ± SD, with statistical significance indicated for between-group comparisons.
