# Supplementary material for: tRF‐15:31‐Arg‐CCT‐3‐1 Improves Diabetic Kidney Disease by Reducing AATF and Alleviating Mesangial Cell Oxidative Stress and Inflammation
Source: Immun Inflamm Dis. 2026 Oct 19;14(10):e70561. doi: 10.1002/iid3.70561 (PMC13678860; doi:10.1002/iid3.70561)
Supplement: Supplementary file 2 [file IID3-14-e70561-s001.docx]

**Supplementary Figure. S1**

**Supplementary Figure. S1** The DKD model was successfully established in C57BL/6J mice at 12 weeks, as shown by blood glucose (**A**) , 24-hour urine volume (**B**), and urinary protein measurements (**C**). ***P < 0.001，ns：no statistical difference.

**Supplementary Figure. S2**

**Supplementary Figure. S2** Blood glucose was measured in each group on the 20-week-old mice (**A**). ***P < 0.001，ns：no statistical difference.

**Supplementary Figure. S3**

**Supplementary Figure. S3** the RBP prediction website：SFRS1 might be the shared RBP for tRF-15:31 and AATF, with a potential binding site of “UGGA”.
